# Herbivore-Mediated Selection on Floral Display Covaries Nonlinearly With Plant-Antagonistic Interaction Intensity Among Primrose Populations

**DOI:** 10.3389/fpls.2021.727957

**Published:** 2021-11-11

**Authors:** Yun Wu, Spencer C. H. Barrett, Xuyu Duan, Jie Zhang, Yongpeng Cha, Chengyi Tu, Qingjun Li

**Affiliations:** ^1^School of Civil Engineering, Architecture and Environment, Xihua University, Chengdu, China; ^2^Yunnan Key Laboratory of Plant Reproductive Adaptation and Evolutionary Ecology, Yunnan University, Kunming, China; ^3^Laboratory of Ecology and Evolutionary Biology, School of Ecology and Environmental Science, Yunnan University, Kunming, China; ^4^Department of Ecology and Evolutionary Biology, University of Toronto, Toronto, ON, Canada; ^5^College of Landscape Architecture, Sichuan Agricultural University, Chengdu, China

**Keywords:** herbivore-mediated selection, nonlinear covariation, opportunity for selection, biotic interaction intensity, *Primula florindae*, spatial variation

## Abstract

Quantifying the relations between plant-antagonistic interactions and natural selection among populations is important for predicting how spatial variation in ecological interactions drive adaptive differentiation. Here, we investigate the relations between the opportunity for selection, herbivore-mediated selection, and the intensity of plant-herbivore interaction among 11 populations of the insect-pollinated plant *Primula florindae* over 2 years. We experimentally quantified herbivore-mediated directional selection on three floral traits (two display and one phenological) within populations and found evidence for herbivore-mediated selection for a later flowering start date and a greater number of flowers per plant. The opportunity for selection and strength of herbivore-mediated selection on number of flowers varied nonlinearly with the intensity of herbivory among populations. These parameters increased and then decreased with increasing intensity of plant-herbivore interactions, defined as an increase in the ratio of herbivore-damaged flowers per individual. Our results provide novel insights into how plant-antagonistic interactions can shape spatial variation in selection on floral traits and contribute toward understanding the mechanistic basis of geographic variation in angiosperm flowers.

## Introduction

A major goal in evolutionary biology is to understand how biotic interactions shape spatial variation in natural selection acting on angiosperm floral traits (Harder and Johnson, 2009; Van der Niet et al., 2014). Spatial variation in selection is an important underlying mechanism of many evolutionary patterns and processes including the maintenance of heritable variation in quantitative traits (Grant and Price, 1981), coevolutionary interactions (Paudel et al., 2016), and the geographical differentiation of floral traits leading to speciation (Siepielski et al., 2013). Numerous studies have shown the critical role of plant-pollinator interactions (mutualists) in contributing to spatial variation in natural selection on floral traits (Chapurlat et al., 2015; Wu and Li, 2017; Wu et al., 2018). In addition to plant-pollinator interactions, plant populations often experience among-population variation in herbivory (antagonists), which can also contribute to spatial variation in natural selection acting on floral traits (Vanhoenacker et al., 2013). Quantifying the relations between the intensity of biotic interactions (plant-pollinator interactions and/or plant-herbivore interactions) and natural selection (pollinator- and/or herbivore-mediated selection) among populations is a key step toward understanding how spatial variation in ecological interactions drives adaptive differentiation and the potential for speciation (Vanhoenacker et al., 2013). Empirical work on natural selection has provided evidence that the relations between biotic interaction intensity, the opportunity for selection (defined as the variance in relative fitness; Downhower et al., 1987; Weis et al., 1992) and the strength of selection are often linear among populations (Weis et al., 1992; Wilson, 1995; Ashman and Morgan, 2004; Moeller and Geber, 2005). However, this may not always be the case.

**FIGURE 1:**
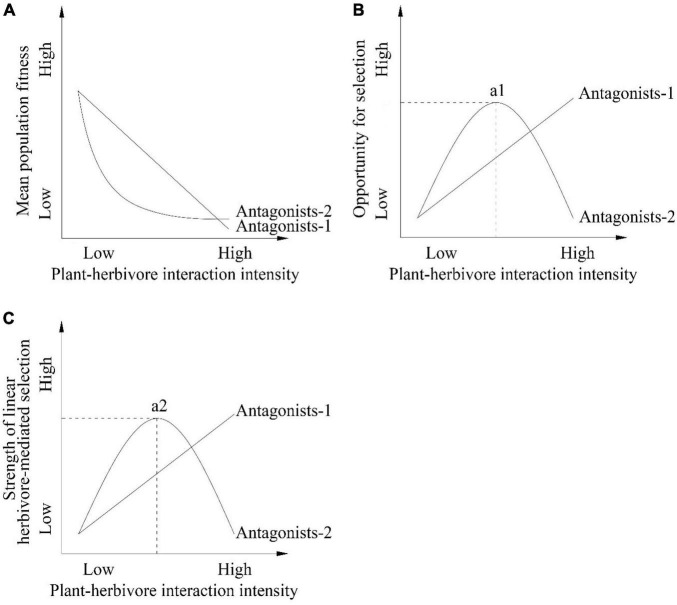
Hypotheses and predictions of the associations between plant-herbivore interaction intensity and **(A)** mean population fitness **(B)** opportunity for selection **(C)** strength of linear herbivore-mediated selection among populations. Mean population fitness **(A)** can decrease with increasing intensity of plant-herbivore interactions or first decrease with the increasing intensity of plant-herbivore interactions and then stabilize when interaction intensity is high among populations because the physiological and biochemical plant defense mechanisms and/or pollinators may reduce the negative effect of herbivores on mean population fitness. In contrast, the opportunity for selection **(B)** and strength of linear herbivore-mediated selection **(C)** can increase with increasing plant-herbivore interaction intensity or first increase with increasing plant-herbivore interaction intensity and then decrease when the intensity of plant-herbivore interactions are high among populations. In the antagonists-1 scenario the effect of herbivores on the host plant are not influenced by other factors. In contrast in the antagonists-2 scenario the effect of herbivores on the host plant are influenced by other factors such as pollinators and/or the physiological and biochemical defense of the host plant. The a1 and a2 points represent the greatest opportunity for selection and the strongest strength of linear herbivore-mediated selection by antagonists-2 prediction, respectively. The dashed lines in **(B,C)** represent the hypothesis of plant-herbivore interaction intensity when there is the greatest opportunity for selection and the strongest strength of linear herbivore-mediated selection.

For antagonistic interactions, such as plant-herbivore interactions, empirical studies have demonstrated herbivore-mediated directional selection on several floral traits within populations, including flowering phenology, floral display, and floral scent traits (Gómez, 2003, 2005; McCall and Irwin, 2006; Sandring et al., 2007; Kessler and Halitschke, 2009; Caruso et al., 2010; Kawagoe and Kudoh, 2010; König et al., 2015; Sletvold et al., 2015; Knauer and Schiestl, 2017). The relations between the intensity of plant-antagonist interactions (e.g., herbivores) and natural selection acting on floral traits imposed by antagonists among populations is expected to be quantifiable because of the strong direct effect of antagonists on absolute plant fitness per individual and mean population fitness. Indeed, herbivores can cause a reduction in mean population fitness (Figure 1A, antagonists-1 scenario) by consuming flowers and fruits or influencing the attractiveness of flowers to pollinators (McCall and Irwin, 2006). An increasing plant-herbivore interaction intensity is expected to increase the variance in relative fitness (opportunity for selection; Figure 1B, antagonists-1 scenario). Empirical studies have demonstrated that the strength of selection on a trait is influenced by the opportunity for selection (Vanhoenacker et al., 2013; Trunschke et al., 2017). As a result, an increasing plant-herbivore interaction intensity is expected to increase the strength of herbivore-mediated selection (Figure 1C, antagonists-1 scenario).

When a large proportion of flowers and seeds of an insect-pollinated host plant are consumed by herbivores, a further increase in the intensity of plant-herbivore interactions may not increase the strength of linear herbivore-mediated selection on floral traits among populations. First, selection mediated by herbivores within some populations will not increase when most flowers and seeds produced by more attractive phenotypes to herbivores are consumed (a depletion effect; Vanhoenacker et al., 2013). Second, high levels of consumption of flowers and seeds by herbivores may also trigger physiological and biochemical defense mechanisms of target plants (Agrawal et al., 2018; Keith and Mitchell-Olds, 2019; Maron et al., 2019; Santangelo et al., 2019; Sercu et al., 2020). These responses have the effect of reducing the negative effect of herbivores on mean population fitness (Figure 1A, antagonists-2 scenario) resulting in a reduction in the opportunities for selection and the strength of linear herbivore-mediated selection on floral traits among populations (Figures 1B,C, antagonists-2 scenario). Third, a large consumption of flowers by herbivores may be expected to reduce the attractiveness of flowers to pollinators and this may incur strong selective pressures from pollinators within some populations. The additive effect of pollinators should reduce the strength of selection mediated by herbivores (Irwin and Brody, 2011). For example, in a *Gymnadenia conopsea* population experiencing one-quarter herbivore intensity (25% of all plants damaged by herbivores), pollinators and herbivores that consumed flowers and developing fruits mediated conflicting selection on flowering phenology (Sletvold et al., 2015). As a consequence, the additive effect from pollinators counteracted the strength of herbivore-mediated selection on this trait.

Collectively, the results of these earlier works suggest that the strength of herbivore-mediated selection should first increase with increasing plant-herbivore interaction intensity, and then decline when the intensity of plant-herbivore interactions becomes greater among populations (Figure 1C, antagonists-2 scenario). This scenario predicts a nonlinear association between the strength of linear herbivore-mediated selection and the intensity of antagonistic interactions among populations. Previous studies have demonstrated interpopulation variation in antagonist-mediated selection on plant reproductive traits (Toju and Sota, 2006; Toju, 2007; Kawagoe and Kudoh, 2010; reviewed in Futuyma and Kirkpatrick, 2017), and in some cases have shown that herbivore-mediated selection on plant reproductive traits exhibits nonlinearity (stabilizing selection) within a population (e.g., Wise and Hébert, 2010). However, knowledge is limited regarding nonlinear associations between the strength of herbivore-mediated selection on floral traits and the intensity of herbivory, especially among multiple populations of plant species (Vanhoenacker et al., 2013).

Here, we test predictions involving linear or nonlinear relations between the intensity of plant-herbivore interactions and the strength of herbivore-mediated selection on floral traits (see Figure 1) in 11 populations of the insect-pollinated primrose (*Primula florindae*) over 2 years using two experimental herbivore treatments. Our initial field observations revealed that the intensity of plant-herbivore interactions varied considerably among the 11 studied populations (Supplementary Table 4). We predicted that herbivores might influence the absolute fitness per plant and mean population fitness through both a direct negative effect on flowers and an indirect negative effect on pollinator visitation. Several studies have provided evidence that floral display traits (Sletvold and Grindeland, 2008; Theis et al., 2014) and flowering phenology (König et al., 2015; Fogelström et al., 2017) separately attract herbivores and influence the intensity of plant-herbivore interactions resulting in herbivore-mediated selection. We experimentally quantified herbivore-mediated selection on three floral traits (two display traits and one phenological trait) within populations and tested the hypothesis that variation in plant-herbivore interaction intensity among populations predicted spatial variation in herbivore-mediated selection on these traits. Our study specifically addressed two key questions: (i) Does the opportunity for selection among populations covary nonlinearly with plant-herbivore interaction intensity? (ii) Does the strength of linear herbivore-mediated selection covary nonlinearly with increasing intensity of plant-herbivore interactions among populations?

## Materials and Methods

### Study Species and Populations

*Primula florindae* is a distylous (populations with long- and short-styled morphs, see Richards, 2003) insect-pollinated perennial herb distributed across alpine regions of southwest China. In common with most distylous species, populations are self- and intramorph incompatible and obligately outbreeding resulting in floral morph ratios that are close to 1:1 in most populations, including those that we studied (Supplementary Table 1). Plants typically produce a basal rosette of leaves and 10 to 30 flowers in a single umbel (Supplementary Figure 1). The flowering period of populations is from June to August with fruiting from August to September, both depending on elevation.

**FIGURE 2:**
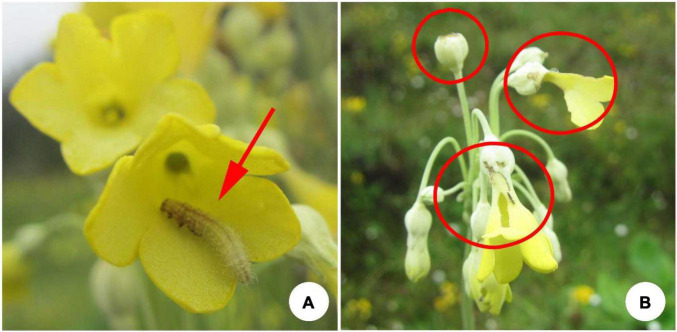
**(A)** The larvae of the brindle plum moth on a flower of *Primula florindae*. **(B)** Damage to flowers of *P. florindae* caused by the larvae of the brindle plum moth.

We conducted experiments in 11 natural populations of *P. florindae* occurring on SeJiLa Mountain, Linzhi County, Xizang, Southwest China (Supplementary Table 2). The populations were located in meadows, ditches or at the forest edge under moist conditions and were separated from one another by topographical barriers, habitat conditions and forest patches. Flowers of *P. florindae* were either damaged or completely destroyed by herbivores in each of the studied populations with the intensity of herbivory varying considerably among populations. Larvae of the brindle plume moth *Amblyptilia punctidactyla* was by far the most abundant herbivore across all populations. From June to August, adults of this moth lay eggs in flower buds of *P. florindae*. After *ca*. 2 days, the larvae hatch and consume the ovary, style and petals to varying degrees, moving among flowers within an inflorescence (Figure 2). In addition, the larvae feed on the seeds of this species.

Because *P. florindae* is self- and intramorph-incompatible, reproductive success depends entirely on pollinator-mediated cross-pollination between the floral morphs. The main pollinators visiting our populations were bumble bees, primarily *Bombus richardis* and *B. convexus*. We predicted that flowers damaged or near destroyed by larvae of *A. punctidactyla* would have reduced attractiveness to bumble bees visiting flowers in populations. We conducted the experiments described below in populations 5, 6, and 9 during 2018 and all remaining populations (1, 2, 3, 4, 7, 8, 10, 11) in 2019.

### Field Experiments and Trait Measurements

During 2018 and 2019, we marked 60–125 pairs of plants within each population based on spatial proximity, for a total of 120–250 separate individuals. Because *P. florindae* does not propagate clonally, the identification of genets (individual plants) was unambiguous. We randomly assigned the two plants per position to one of the two herbivory treatments: herbivore-present treatment (HP) and herbivore-exclusion treatment (HE). All 11 populations were visited every 2 days throughout the flowering period in 2018 and 2019, respectively. For individual populations this averaged approximately 6 weeks. During each visit, we checked all open flowers in the HE treatment and herbivores were manually removed from plants; thus herbivory was close to zero in all populations for the HE treatment (see section “Results”).

We recorded the start of flowering (Julian day, day of year) for each individual as the day when the first flower fully opened. For the first three open flowers on each individual, we measured corolla size (maximum diameter of corolla), to the nearest 0.01 mm using digital calipers, and used mean corolla size for each individual for the subsequent analysis. We also recorded the total number of flowers produced by each individual at the end of the flowering period.

To quantify the female reproductive success of plants, we recorded the number of fruits at maturation and these were collected to determine the number of seeds per fruit. Total seed production per plant was then obtained for all sampled plants. In all populations at the end of the flowering period we recorded the number of flowers that had experienced herbivory for each individual in both the HP and HE treatments. We defined herbivory as the removal of tissue from the sepals or floral parts. Although some flowers in an umbel had wilted petals at the end of flowering, no flowers had fallen from umbels. We therefore estimated the intensity of herbivory for each individual as the number of flowers showing herbivory divided by the total number of flowers produced by the plant.

### Statistical Analysis

We tested the effects of population, herbivory treatment and their interaction on each floral trait (flowering start date, number of flowers and corolla size per plant), intensity of herbivory and female reproductive success (fruit production, seeds per fruit, and seeds per plant) by using multiple two-way analysis of variance (ANOVA) models. To improve normality of data, data for flowering start date, number of flowers, corolla size, fruit production, seeds per fruit and seeds per plant were log_10_ transformed, and data for herbivory intensity was square-root transformed prior to ANOVA analyses.

### Selection Gradient Analysis

Following the methods of Lande and Arnold (1983), we estimated selection gradients from the multiple regression models separately for treatments in each population. In the regression models, we used the relative number of seeds per plant and the three standardized floral traits (flowering start date, number of flowers, and corolla size) as the response variable and explanatory variables, respectively. For each population and treatment combination, we calculated relative number of seeds per plant by dividing individual seeds per plant by the population mean and trait values were standardized to a mean of 0 and a variance of 1 (both using the original data). We initially included linear and quadratic (γ_*ii*_) terms to quantify stabilizing or disruptive selection. Few quadratic selection gradients were statistically significant and the variance inflation factors (VIFs) of some quadratic terms were >5.0, indicating that there was multicollinearity (Quinn and Keough, 2002). To limit model complexity, we only include the linear terms to estimate the directional selection gradients (β_*i*_) in the present study. To test for multicollinearity in these linear regression models, we calculated the VIFs for the linear terms. All VIFs were <1.8, indicating that there was no multicollinearity (Quinn and Keough, 2002).

### Variation Among Populations in Herbivore-Mediated Selection

To test whether herbivore-mediated selection varied among populations, we included the data from the plants of both the HP and HE treatments in the 11 populations using analysis of covariance (ANCOVA). The model included the relative number of seeds per plant as a response variable and the three standardized traits, population (11 populations), treatment (HP vs. HE), trait × population, trait × treatment and trait × population × treatment interactions as explanatory variables. Significant trait × population × treatment interaction indicated that herbivore-mediated selection varied among the populations. Because some trait × treatment interaction terms were significant, we only analyzed the data from the HP treatment plants using ANCOVA to test whether net directional selection varied among populations. The model included the relative number of seeds per plant as the response variable and our three standardized traits (flowering start date, number of flowers, and corolla size), the population and the trait × population interactions as explanatory variables. To quantify herbivore-mediated selection, we subtracted the estimated selection gradients for each trait for plants that were subjected to the herbivore-exclusion treatment (β_*HE*_) from the estimates obtained for plants under the herbivory present treatment (β_*HP*_; Δβ_herb_ = β_*HP*_–β_*HE*_) with its associated standard error $\sqrt{S\,E_{\beta_{H\,P}}^{2}+S\,E_{\beta_{H\,E}}^{2}}$ (Chapurlat et al., 2019). To test whether herbivore-mediated selection on each floral trait within each population was significant, we included the data in the ANCOVA from the plants of both the HP and HE treatments separately for each population. The model included the relative number of seeds per plant as a response variable and the three standardized traits, treatment and trait × treatment as explanatory variables.

### Relations Between Herbivore Intensity, Opportunity for Selection and Strength of Linear Herbivore-Mediated Selection Among Populations

We separately estimated the opportunity for selection as the variance in the relative number of seeds per plant for each treatment and population. In our study, the opportunity for selection was estimated without sampling error because we measured seed production for all individuals in each population. In addition, our estimates of herbivory intensity were fairly precise (low sampling error relative to the variability in herbivory intensity from any one population) among populations. As a consequence, we used the ordinary least squares (OLS) regression model to test whether the associations between the opportunity for selection and herbivory intensity among populations were linear or nonlinear (models A and B). In model A, we used the opportunity for selection as the response variable and the linear term of the herbivory intensity as the explanatory variable. In model B, we used the opportunity for selection as the response variable and the linear and quadratic terms of the herbivory intensity as explanatory variables. We used the original data (not transformed) of herbivory intensity in these models. These models were estimated for each treatment. We calculated the Akaike information criterion (AIC) for each model hypothesis. The lowest AIC value of the model fit the data best (Akaike, 1974). We selected the best model (see section “Results”) and estimated its *P*-value.

To quantify the nonlinear associations between herbivory intensity and the strength of herbivore-mediated selection among populations, we first estimated the linear herbivore-mediated selection gradients of floral traits within a population (see above) and then fitted these estimates with a quadratic regression model among populations. Because herbivore-mediated selection gradients were estimated in our study, we used Bayesian models (MCMCglmm function in the R package; Hadfield, 2010) which account for sampling error of this variable (Morrissey, 2016; Hunter et al., 2018) to test whether the associations between herbivory intensity and the strength of linear herbivore-mediated selection are linear or nonlinear (models C and D). For MCMCglmm models, the character vector of trait distributions was normally distributed. The random effects are viewed as an approximation to the expected standard deviation of the measurement error. Therefore, we define a diagonal matrix with the standard errors on the diagonal. The probabilistic distribution of residual errors is the standard normal distribution. In these models, we used only the herbivory intensity data from the HP treatment plants because they represented natural levels of herbivory. In addition, our estimates of herbivory intensity were fairly precise with low sampling error relative to the variability in herbivory intensity from any one population. Also, the sampling error of herbivory intensity was much smaller than the strength of herbivore-mediated selection among populations. Taking this and the algorithm of MCMCglmm function into consideration, we used Bayesian models in our study, which take account of the sampling error of the response variable (see Morrissey, 2016). Because some of the herbivore-mediated selection gradients had negative values, we calculated their absolute value for each trait and each population by following the methods of Morrissey (2016). We also re-calculated the sampling error of the absolute value of herbivore-mediated selection gradients from the variance of a folded normal distribution, also following Morrissey (2016). In model C, we used the absolute value of herbivore-mediated selection gradients as the response variable and the linear term of the herbivory intensity as the explanatory variable. In model D, we used the absolute value of herbivore-mediated selection gradients as the response variable and the linear and quadratic terms of herbivory intensity as explanatory variables. The Bayesian models were estimated separately for each floral trait. We calculated the Deviance information criterion (DIC) for each Bayesian model. The lowest DIC value of the model fit the data best (Berg et al., 2004). We selected the best model (see section “Results”) in the present study. We performed the analyses with R 3.6.1 (R Core Team, 2019) and used it to generate graphs.

## Results

### Variation in Floral Traits, Herbivore Intensity, Reproductive Success and Opportunity for Selection Among Populations

None of the floral traits that we measured differed significantly (*P* > 0.05) between the HP and HE treatments within each population, but all floral traits varied significantly among populations (Supplementary Table 3).

The intensity of herbivory varied among populations (*F*_10,18__12_ = 37.1, *P* < 0.001) and between treatments (*F*_1,1812_ = 4529.07, *P* < 0.001; Supplementary Tables 3, 4). In the HP treatment, herbivory intensity ranged from 0.154 ± 0.156 (mean ± SD) to 0.557 ± 0.221 from population 1 to 11. The HE treatment successfully excluded herbivores and as expected herbivory was near zero in all populations (Supplementary Table 4).

Fruit production, seeds per fruit and seeds per plant varied among populations and between treatments (*P* < 0.001; Supplementary Tables 3, 4). Plants in population 1 produced more fruits, more seeds per fruit and had higher total seeds per plant than in other populations for both the HP and HE treatments (Supplementary Table 4). Overall, plants produced more fruits, more seeds per fruit (except for the population 3) and more seeds per plant in the HE treatment than in the HP treatment.

For the HP treatment, the greatest opportunity for selection was recorded in population 10 and the lowest was observed in population 2. For the HE treatment, the greatest opportunity for selection was recorded in population 4 and the lowest in population 5.

### Phenotypic Selection Within Populations

**FIGURE 3:**
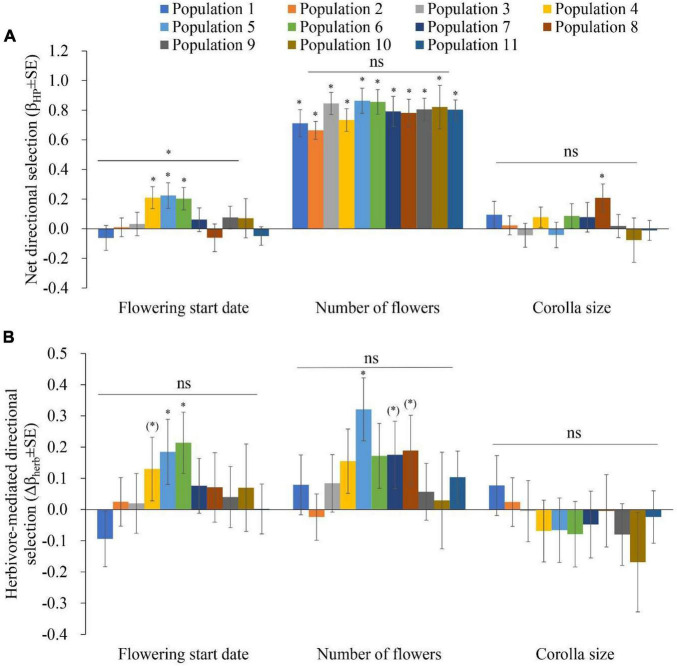
Phenotypic selection in 11 populations of *Primula florindae*: **(A)** Net directional selection gradients (β_*HP*_ ± SE) and **(B)** herbivore-mediated directional selection gradients (Δβ_herb_ ± SE) related to the flowering start date, number of flowers and corolla size among populations. Symbols above individual bars indicate the level of significance of the selection gradients. Symbols above the lines indicate whether directional selection varied among the populations, as indicated by the significant trait × population and trait × treatment × population terms in the ANCOVAs. ^∗^*P* < 0.05; ^(*)^*P* < 0.1; and ^ns^*P* > 0.1.

Net directional selection on the start of flowering varied among populations, as indicated by the significant flowering start × population interaction (*F*_10,920_ = 2.137, *P* = 0.02) obtained through ANCOVA (Supplementary Table 5 and Figure 3A). A later flowering start date was selected in populations 4, 5, and 6 (Figure 3A and Supplementary Table 6). There was directional selection for higher flower number in all 11 populations (Figure 3A and Supplementary Table 6). Larger corolla sizes were selected in population 8 (Figure 3A and Supplementary Table 6).

Herbivore-mediated selection on flowering start date was detected in populations 4 (marginally significant, Δβ_herb_ ± SE = 0.13 ± 0.102, *P* = 0.094), 5 and 6 (Figure 3B, Supplementary Figures 2A,B,C, and Supplementary Table 6). Herbivore-mediated selection on number of flowers per plant was detected in populations 5, 7 (marginally significant, Δβ_herb_ ± SE = 0.175 ± 0.108, *P* = 0.089) and 8 (marginally significant, Δβ_herb_ ± SE = 0.189 ± 0.113, *P* = 0.078; Figure 3B, Supplementary Figures 2D,E,F, and Supplementary Table 6). Significant herbivore-mediated selection on corolla size was not detected in any population.

### Relations Between Herbivory Intensity, the Opportunity for Selection and the Strength of Herbivore-Mediated Selection Among Populations

**FIGURE 4:**
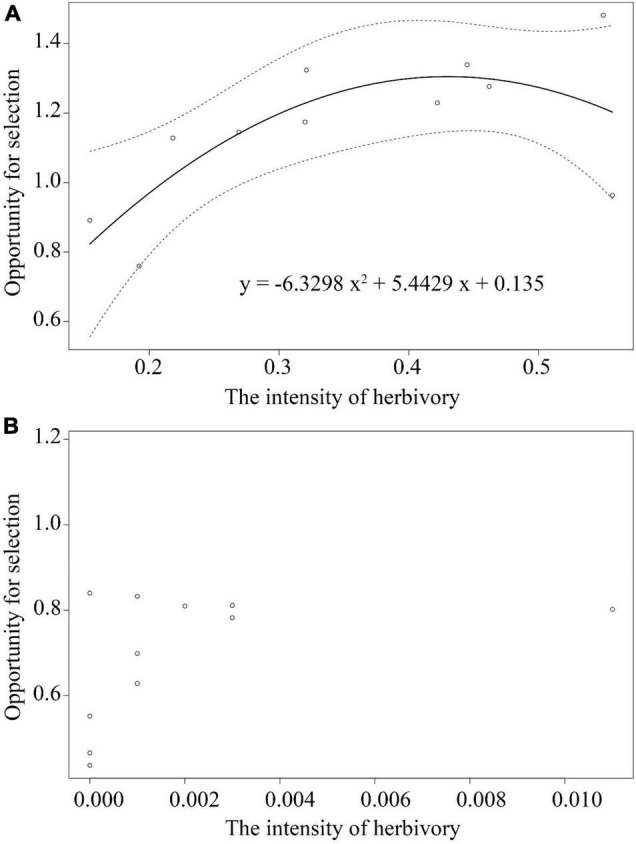
Nonlinear relations between the opportunity for selection and herbivory intensity among the herbivore present treatment plants **(A)** and among the herbivore exclusion treatment plants **(B)** among 11 populations of *Primula florindae*. The best-fit model for HP treatment is provided. The dashed lines represent the 95% confidence interval of the fitted curves.

Goodness-of-fit statistics for the models indicated that B and D (quadratic regression models, see Supplementary Table 7) had the lowest AIC and DIC values (except for corolla size) and thus had a better fit to our data. There was no association between the opportunity for selection and herbivory intensity in the HE-treated plants (Supplementary Table 7 and Figure 4B). In contrast, the opportunity for selection first increased and then decreased with increasing herbivory intensity among populations in plants of the HP treatment (*y* = −6.3298x^2^ + 5.4429x + 0.135; Supplementary Table 7, Figure 4A).

**FIGURE 5:**
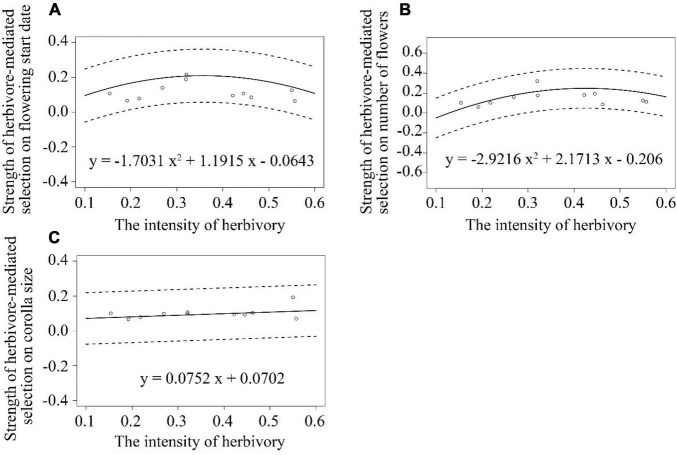
Associations between the strength of herbivore-mediated selection on: **(A)** flowering start date **(B)** number of flowers **(C)** corolla size per plant and the intensity of herbivory among 11 populations of *Primula florindae*. The best-fit models for each trait are also provided. The dashed lines represent the 95% confidence interval of the fitted curves.

The strength of herbivore-mediated selection on number of flowers per plant first increased and then decreased with an increasing intensity of herbivory among populations, as indicated by the significant negative quadratic regression model (*y* = −2.9216x^2^ + 2.1713x–0.206; Supplementary Table 7 and Figure 5B). There were no associations between the strength of herbivore-mediated selection on flowering start and herbivory intensity or between the strength of herbivore-mediated selection on corolla size and herbivory intensity (Supplementary Table 7 and Figures 5A,C).

## Discussion

Our experimental study of phenotypic selection in 11 populations of an alpine primrose demonstrated herbivore-mediated directional selection on floral display and flowering time. The results also indicated that the opportunity for selection and the strength of herbivore-mediated selection on floral display covaried nonlinearly with an increasing intensity of plant-herbivore interactions among populations. Our results support the overall hypothesis that variation in the intensity of plant-antagonistic interactions has the potential to drive spatial variation in natural selection on floral traits.

### Herbivore-Mediated Selection on Floral Traits Within Populations

We provide evidence for herbivore-mediated selection on the number of flowers and the start of flowering in our studied populations. Our results are consistent with several earlier studies reporting that floral display traits and flowering phenology separately attract herbivores influencing plant-herbivore interaction intensity and thus are subject to selection (Theis et al., 2014; Fogelström et al., 2017). However, the occurrence and strength of herbivore-mediated selection differed among the traits we investigated.

For insect-pollinated plants, larger floral displays are predicted to promote pollinator attraction thus increasing pollen export and receipt (Caruso et al., 2003; Sandring and Ågren, 2009). Furthermore, larger displays are likely to be more attractive to floral herbivores because these visitors may use the same cues to locate and interact with flowers (Strauss and Irwin, 2004). More flowers per plant would be expected to attract a greater number of herbivores because of the opportunity for a greater number of feeding targets, thus contributing to herbivore-mediated selection. Our results indicated that herbivore-mediated selection on the number of flowers can partially explain the net selection on this trait. This suggests that other agents in addition to pollinators will also generate selective pressures for greater flower production.

It is important to recognize that whether phenotypic selection can change trait distributions between generations in a population is dependent on the occurrence of standing genetic variation for individual traits (Conner and Hartl, 2004). A survey of studies on phenotypic selection in floral traits reported that flower production, a component of floral display, experienced phenotypic selection three time more often than any other floral traits that were examined (Harder and Johnson, 2009). This result is not unexpected because flower number per plant is a highly plastic trait that is strongly influenced by the resource status of individuals which can vary dramatically in heterogeneous environments (Gottlieb, 1977; Harper, 1977). Despite this phenotypic variation a survey of the heritability of floral and inflorescence traits among species revealed a moderate heritability (*h*^2^ = 0.34) of flower number and display (Ashman and Majetic, 2006). This finding indicates opportunities for genetic responses to selection and future heritability studies of flower production in *P. florindae* populations would be useful to determine the extent to which selection responses imposed by biotic agents can have evolutionary consequences.

Previous studies have demonstrated the potential role of biotic (e.g., mutualists, antagonists) and abiotic agents (e.g., temperature, rainfall) in driving the evolution of flowering phenology (Cleland et al., 2007; Elzinga et al., 2007). Our results indicated that herbivore-mediated selection on the start of flowering could account for most of the net selection (|Δβ_herb_|/|β_*HP*_|, exceeding 62% in 9 of 11 studied populations) on this trait. This result is consistent with the hypothesis that herbivores can be important agents of selection on flowering phenology. The abundance of herbivores and their phenotypic preferences are predicted to cause selection on floral phenological traits (Cornelissen and Stiling, 2005). In our populations, *A. punctidactyla* is abundant from mid-July to August. A later date for the start of flowering is therefore likely to coincide with the activity of herbivores. Elzinga et al. (2007) reviewed the relation between flowering phenology and biotic interaction and found that mutualists tended to favor peak or earlier flowering, but in contrast, antagonists tended to favor off-peak or later flowering. Our results only partially supported this prediction as herbivores only favored later flowering in our study.

We detected no evidence for significant herbivore-mediated selection on corolla size in the *P. florindae* populations we investigated. However, our results demonstrated selection for larger corolla size in the HE treatment in population 8, suggesting that other agents (e.g., pollinators) may have mediated selection on this trait. Larger flower sizes may increase the amount of pollen imported and exported because larger flowers can be more attractive to pollinators (Worley and Barrett, 2000; Benitez-Vieyra et al., 2006; Sánchez-Lafuente and Parra, 2009). In our study, we did not quantify the potential effect of pollinators in shaping floral evolution of *P. florindae* through phenotypic selection analysis. Additional manipulative experiments would be required to determine whether pollinators generate selective pressures on flower-size variation in the presence vs. absence of herbivores.

Interestingly, our results do not conform to the expected negative relation between flower size and number resulting from resource trade-offs (reviewed in Ashman and Majetic, 2006; Caruso, 2006). Our study indicated that natural selection through the antagonistic interaction increased the number of flowers but did not change corolla size. It is not known why this relation was not found in our study populations but the evidence for trade-offs between flower size and number is mixed in the literature (see Table 1 in Worley and Barrett, 2000). Some studies have demonstrated a negative relation (e.g., Sargent et al., 2007) whereas others have not, in some cases reporting positive relations at the species level (e.g., Worley et al., 2000). Several explanations may explain why we failed to detect a negative relation between flower size and number. It is possible that the within-season time scale of our study limited opportunities for long-term selective responses based on trade-offs. Other explanations include the possibility that flower size and number do not directly compete for resources, or that because of the perennial life history of *P. florindae* any trade-offs occur with total life-time flower production not inflorescence flower number.

Herbivore-mediated selection on floral traits was inconsistent among the traits which we investigated, as reported by several other authors (Sandring et al., 2007; Kessler and Halitschke, 2009; Sletvold et al., 2015). The trait preferences of herbivores may contribute to the variation we observed. In our study populations, herbivores preferred a greater flower production and a later flowering start date. The variation in these two traits would influence the intensity of herbivory, whereas corolla size did not. Undoubtedly, the ecological context of each population also contributed toward the variation in selection, as discussed below.

### Nonlinear Relations Between Plant-Herbivore Interaction Intensity, Opportunity for Selection and Strength of Herbivore-Mediated Selection

Biotic interactions can be drivers of the opportunity for selection across populations and the relations between the intensity of biotic interaction and opportunity for selection may in some cases be nonlinear (Benkman, 2013). For example, the opportunity for selection via seeds per fruit declines and then increases with an increasing intensity of plant-pollinator interaction among *Sabatia angularis* populations (Emel et al., 2017). However, relatively few studies have directly estimated the effect of antagonistic interaction strength on the opportunity for selection.

Our results demonstrated that the intensity of plant-herbivore interactions on the opportunity for selection was nonlinear among populations. The opportunity for selection increased and then decreased with an increasing intensity of plant-herbivore interactions. A low intensity of herbivory largely reduced the mean population maternal fitness (seeds per plant) whereas a high intensity of plant-herbivore interactions did not result in greater absolute fitness cost per individual (Supplementary Table 2). These patterns contribute to the nonlinear relations between the opportunity for selection and plant-herbivore interaction intensity among populations (as predicted in Figure 1B, antagonists-2 scenario).

The strength of selection on a trait is influenced by the opportunity for selection and the correlation between the trait and fitness (Vanhoenacker et al., 2013; Trunschke et al., 2017). We found that the strength of herbivore-mediated selection among populations on the number of flowers per plant covaried nonlinearly with the intensity of plant-herbivore interactions (as predicted in Figure 1C, antagonists-2 scenario). In the 11 populations of *P. florindae*, we found a negative quadratic relation between the opportunity for selection and the intensity of herbivory. The strength of herbivore-mediated selection exhibited a similar trend to the opportunity for selection across populations with increasing biotic interaction intensity. A low intensity of plant-herbivore interactions will increase the strength of herbivore-mediated selection because of the increasing opportunity for selection, whereas a high biotic interaction intensity can limit the opportunity for selection. A high plant-herbivore interaction intensity is expected to result in greater absolute fitness cost per individual, thus reducing the variance in relative female fitness and reducing the strength of selection. Indeed, our results for *P. florindae* indicated that when the intensity of herbivory was *ca.* 0.35, the opportunity for selection and the strength of herbivore-mediated selection on the number of flowers per plant declined. As a result, the relations among populations between the opportunity for selection and plant-herbivore interaction intensity, and also between the strength of herbivore-mediated selection and plant-herbivore interaction intensity was nonlinear.

The severity of damage (totally destroyed flowers vs. partially damaged flowers) may have some consequences for the strength of selection. However, in our field surveys we did not differentiate between totally destroyed and partially damaged flowers because of logistical considerations associated with the large sample sizes within each population. Based on our own observations, the severity of damage differed among populations and this source of variation may have contributed to the observed spatial variation in the strength of herbivore-mediated selection on floral traits.

Diverse ecological and demographic factors probably contributed to variation in the patterns of antagonistic selection we observed among populations of *P. florindae*. For example, co-flowering plant species may influence the abundance of brindle plume moth larvae feeding on *P. florindae* populations, especially given that this species is a generalist herbivore. Our observations indicated that larvae of this species also attacked the flowers and fruits of co-occurring plants of *P. alpicola* at some of our study sites, but not to the extent we observed in our focal species because of the more limited abundance of *P. alpicola*. Nevertheless, the variation in antagonistic selection that our study revealed seems likely to be strongly influenced by plant community context.

Our investigations of phenotypic selection only measured plant fitness through maternal reproductive components (fruit and seed set). Previous studies have shown that the strength of selection through male function can be stronger than through female function (e.g., Benitez-Vieyra et al., 2006; Hodgins and Barrett, 2008; La Rosa and Conner, 2017). To comprehensively understand spatial variation in herbivore-mediated selection among populations, male fitness components (e.g., outcrossed male siring success), would be desirable for the quantification of total reproductive fitness. Also, our study only measured herbivore-mediated selection in each primrose population during a single flowering season. For perennial herbs, herbivory experienced in 1 year may affect plant fitness in subsequent years and thus measurements of herbivore-mediated selection within a population across years is necessary to understand the longer-term fitness consequences of this form of biotic interaction.

Our results on a single flowering plant species lead to the fundamental question of how biotic interactions are generally related to the strength of selection in other organisms. The strength of selection is mainly influenced by the intensity of biotic interactions and the fitness-trait covariance (Mezquida and Benkman, 2014). When the relations between fitness and traits are consistent in plants, the strength of selection should covary with the intensity of biotic interactions (Benkman, 2013; Vanhoenacker et al., 2013). However, numerous aspects of the demography of populations and the environmental context in which they occur influence the strength of species interactions (Vanhoenacker et al., 2009; Benkman et al., 2010; Mezquida and Benkman, 2014).

Mobile organisms such as animals may rapidly react to changes in the biotic and abiotic environment thus limiting opportunities for persistent year-to-year patterns of natural selection on traits. The great diversity in organismal life histories and behaviors and the varied ecological contexts in which organisms occur present a major challenge for determining general associations between biotic interaction intensity and the strength of selection. To refine our understanding of this association it is important to not only determine the agents of selection, but also to understand how these vary spatially and cause geographical variation in the nature of selection among populations. Our results have revealed that the opportunity and strength of herbivore-mediated selection covary nonlinearly with the increasing intensity of plant-herbivore interactions. This finding implies that variation in herbivory across flowering plant populations has the potential to cause complex patterns of population differentiation in reproductively important traits.

## Data Availability Statement

The datasets presented in this study can be found in online repositories. The names of the repository/repositories and accession number(s) can be found below: https://datadryad.org/stash/share/-caj8jh_xs3CsOpLBRUQdWByqr3pW2VE3nC_lgUrOmM.

## Author Contributions

YW and QL conceived and designed the study. YW, XD, JZ, and YC conducted the field experiments. YW, CT, and QL analyzed the data. YW, SB, and QL interpreted results and wrote the manuscript. All authors contributed to the article and approved the submitted version.

## Conflict of Interest

The authors declare that the research was conducted in the absence of any commercial or financial relationships that could be construed as a potential conflict of interest.

## Publisher’s Note

All claims expressed in this article are solely those of the authors and do not necessarily represent those of their affiliated organizations, or those of the publisher, the editors and the reviewers. Any product that may be evaluated in this article, or claim that may be made by its manufacturer, is not guaranteed or endorsed by the publisher.
