# Supplementary material for: Herbivore-Mediated Selection on Floral Display Covaries Nonlinearly With Plant-Antagonistic Interaction Intensity Among Primrose Populations
Source: Front Plant Sci. 2021 Nov 11;12:727957. doi: 10.3389/fpls.2021.727957 (PMC8636000; doi:10.3389/fpls.2021.727957)
Supplement: Supplementary file 1 [file Data_Sheet_1.docx]

**Supplementary materials**

**Figures**

**
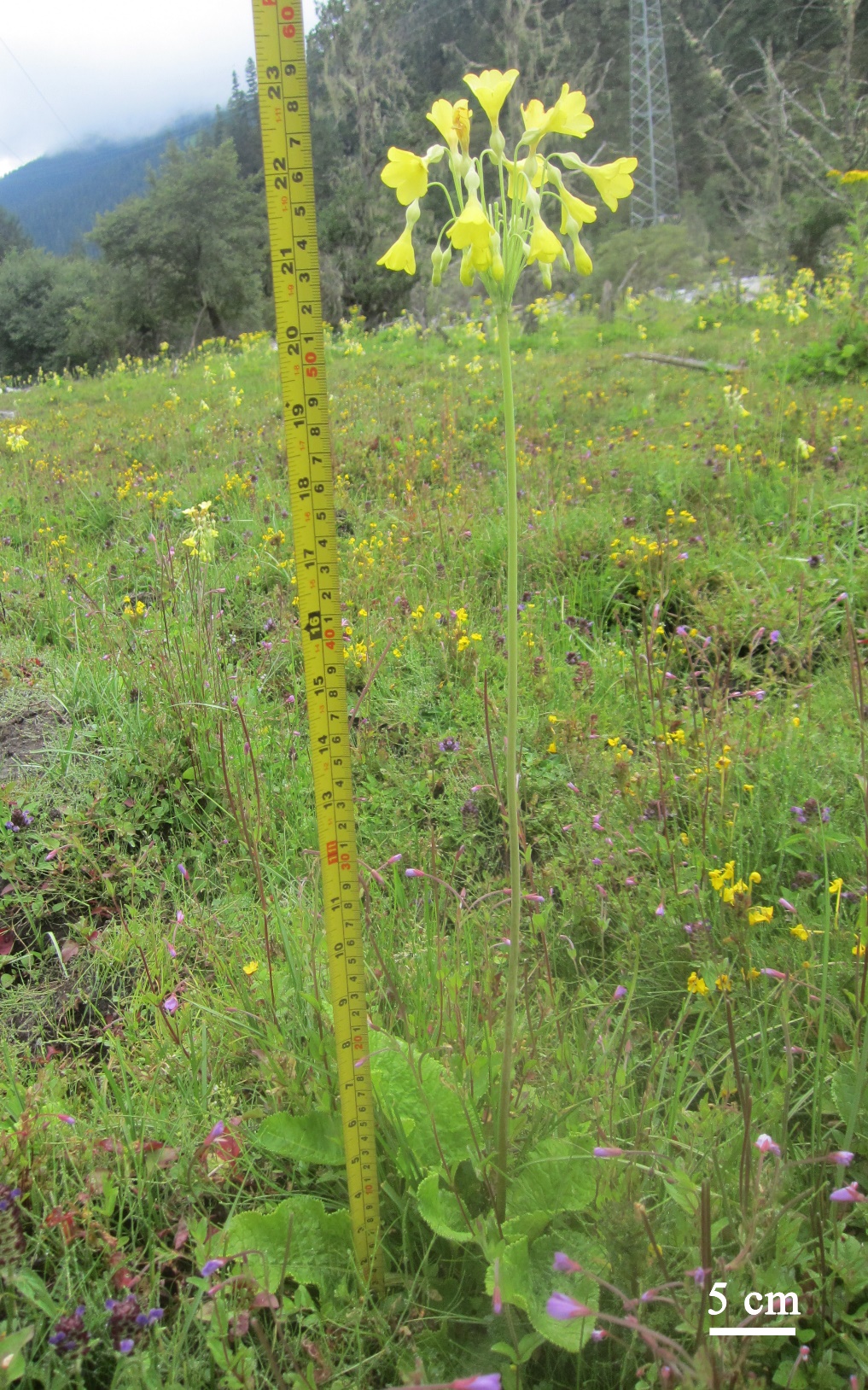
**

**FIGURE S1** The inflorescence of *Primula florindae*.


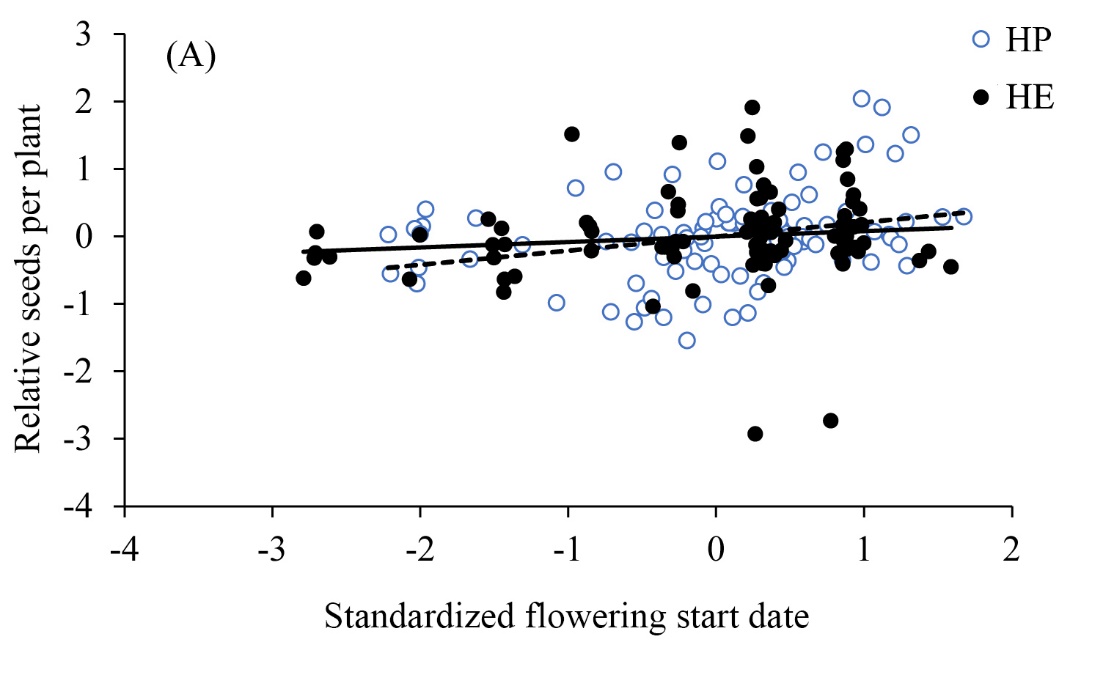


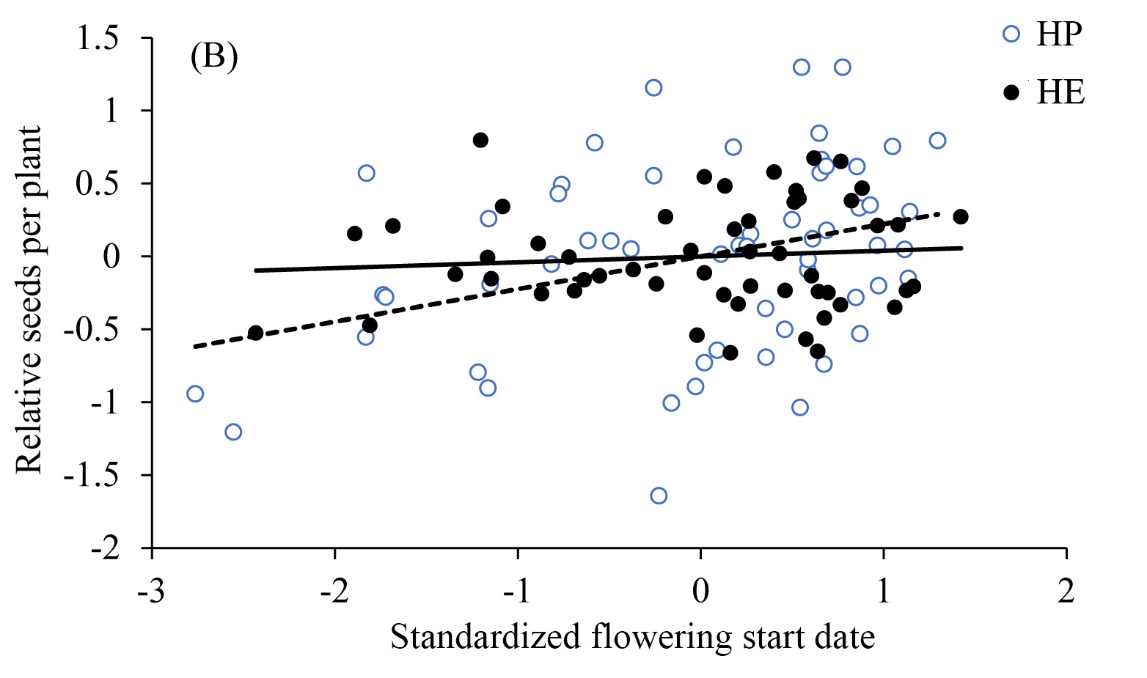


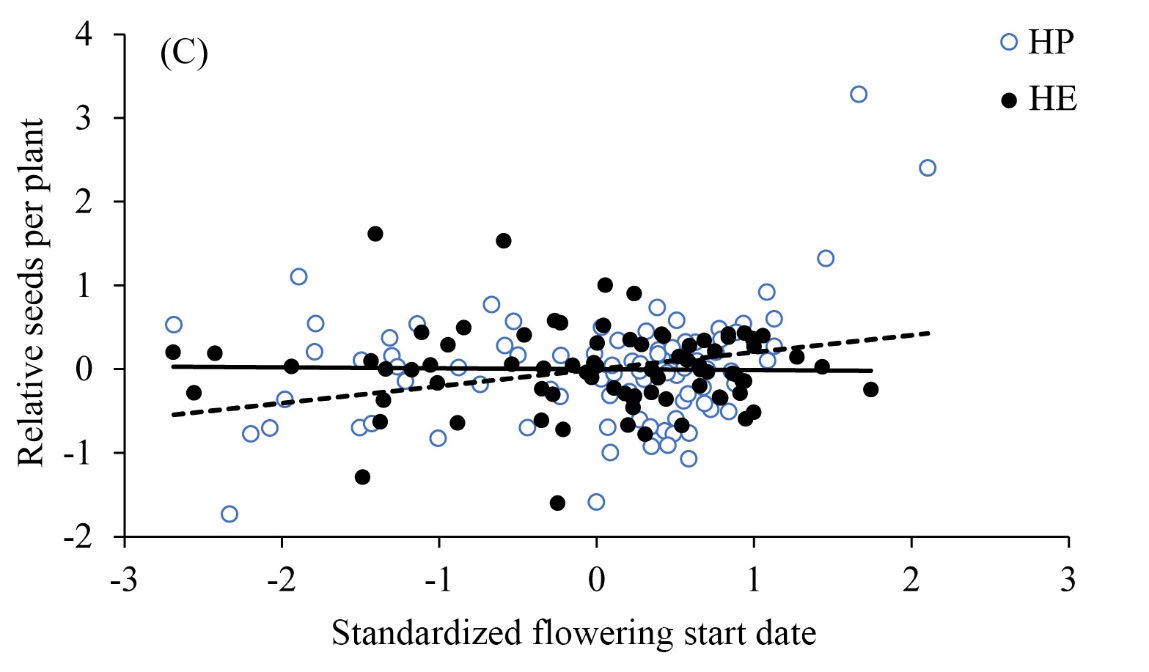


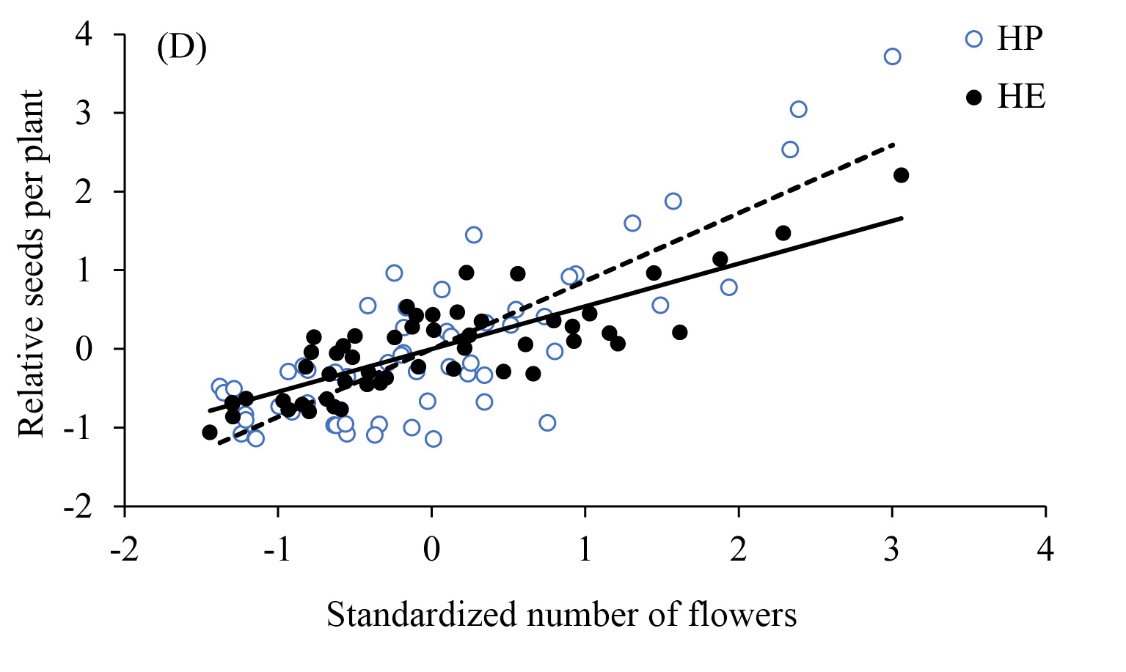


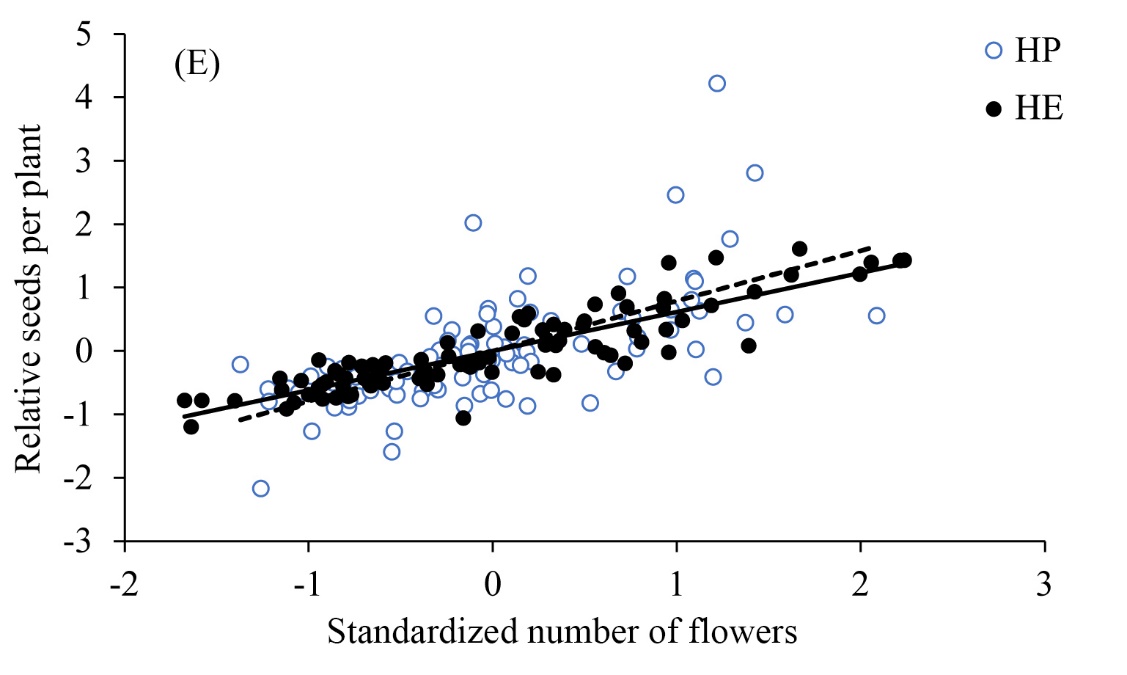


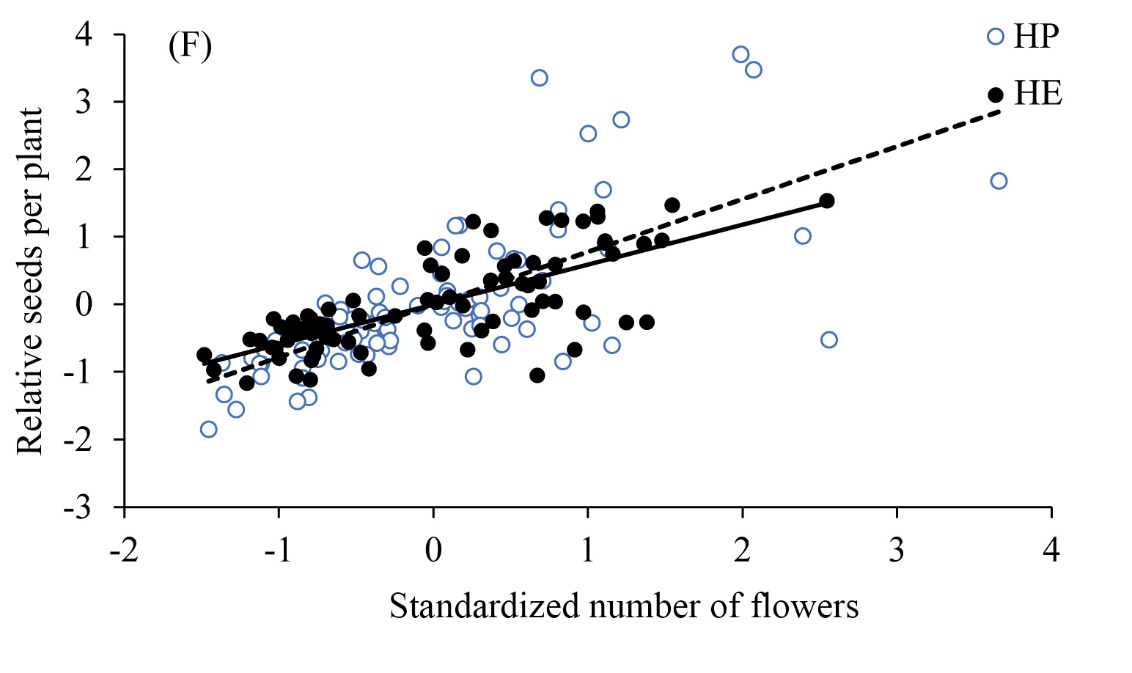


**FIGURE S2** Standardized linear phenotypic selection gradients for flowering start date and number of flowers in herbivore-present plants (HP, open circles, dashed line) and in herbivore-exclusion plants (HE, closed circles, solid line) in population 4 (A), 5 (B and D), 6 (C), 7 (E) and 8 (F) of *Primula florindae*. The selection gradients are illustrated with added-variable plot, in which the residuals from a linear regression model of relative seeds per plant on all traits (except the focal trait) are plotted against the residuals from a regression model of the focal trait on the other traits.

**Tables**

**TABLE S1** Floral morph ratios in 11 populations of *Primula florindae* with *G*-tests for goodness-of-fit of morph ratios to 1:1 within populations. L- and S-morph refer to the long- and short-styled morphs, respectively. * *P* < 0.05; ** *P* < 0.01.

| Population | Number of plants sampled | Frequency of the L-morph | Frequency of the S-morph |  | *G*-statistic |
| --- | --- | --- | --- | --- | --- |
| 1 | 137 | 0.42 | 0.58 |  | 3.232 |
| 2 | 184 | 0.61 | 0.39 |  | 8.766** |
| 3 | 165 | 0.5 | 0.5 |  | 0.006 |
| 4 | 190 | 0.51 | 0.49 |  | 0.021 |
| 5 | 110 | 0.59 | 0.41 |  | 3.657 |
| 6 | 167 | 0.5 | 0.5 |  | 0.006 |
| 7 | 178 | 0.54 | 0.46 |  | 1.102 |
| 8 | 166 | 0.45 | 0.55 |  | 1.545 |
| 9 | 217 | 0.53 | 0.47 |  | 1.038 |
| 10 | 123 | 0.57 | 0.43 |  | 2.357 |
| 11 | 176 | 0.51 | 0.49 |  | 0.023 |
|  |  |  |  | total *G* | 21.753* |
| pooled | 1813 | 0.52 | 0.48 | pooled *G* | 2.94 |
|  |  |  |  | heterogeneity *G* | 18.811* |

**TABLE S2** The geographic location information of the 11 studied *Primula florindae* populations.

| Population | Longitude | Latitude | Elevation |
| --- | --- | --- | --- |
| 1 | 94°44.5369′ E | 29°46.698′ N | 3339 m a.s.l |
| 2 | 94°43.9991′ E | 29°46.3556′ N | 3405 m a.s.l |
| 3 | 94°44.5305′ E | 29°46.4646′ N | 3335 m a.s.l |
| 4 | 94°44.473′ E | 29°46.6841′ N | 3399 m a.s.l |
| 5 | 94°44.5437′ E | 29°46.4558′ N | 3344 m a.s.l |
| 6 | 94°44.4935′ E | 29°46.6685′ N | 3352 m a.s.l |
| 7 | 94°43.9862′ E | 29°45.9478′ N | 3354 m a.s.l |
| 8 | 94°43.7883′ E | 29°46.0577′ N | 3380 m a.s.l |
| 9 | 94°44.5719′ E | 29°46.6294′ N | 3300 m a.s.l |
| 10 | 94°43.9468′ E | 29°45.9578′ N | 3398 m a.s.l |
| 11 | 94°44.5727′ E | 29°46.6578′ N | 3328 m a.s.l |

**TABLE S3** Effects of population and herbivory exclusion treatments (HP vs. HE) on floral traits, herbivory intensity and female reproductive success in *Primula florindae* analyzed with two-way ANOVA.

| Traits, herbivory and fitness | Population |  |  | Treatment |  |  | Population × Treatment | |
| --- | --- | --- | --- | --- | --- | --- | --- | --- |
|  | *F*_10,1812_ | *P* |  | *F*_1,1812_ | *P* |  | *F*_10,1812_ | *P* |
| Flowering start date | 3134.787 | **< 0.001** |  | 1.838 | 0.175 |  | 1.289 | 0.231 |
| Number of flowers | 8.346 | **< 0.001** |  | 0.128 | 0.720 |  | 1.208 | 0.281 |
| Corolla size | 16.288 | **< 0.001** |  | 0.022 | 0.882 |  | 1.724 | 0.070 |
| Herbivory intensity | 37.100 | **< 0.001** |  | 4529.070 | **< 0.001** |  | 31.830 | **< 0.001** |
| Fruit production | 9.565 | **< 0.001** |  | 193.959 | **< 0.001** |  | 1.323 | 0.212 |
| Seeds per fruit | 4.326 | **< 0.001** |  | 79.969 | **< 0.001** |  | 2.824 | **0.002** |
| Seeds per plant | 7.037 | **< 0.001** |  | 160.817 | **< 0.001** |  | 2.052 | **0.025** |

**TABLE S4** Floral traits, herbivory intensity, female reproductive success and opportunity for selection (mean ± SD) for plants in 11 *Primula florindae* populations. HP, herbivore-present treatment; HE, herbivore-exclusion treatment. Sample sizes are provided in the first column.

| Population | Sample size | |  | Flowering start date (Julian date, day of year) | |  | Number of flowers | |  | Corolla size (mm) | |  | Herbivory intensity | |  | Fruit production | |  | Seeds per fruit | |  | Seeds per plant | |  | Opportunity for selection | |
| --- | --- | --- | --- | --- | --- | --- | --- | --- | --- | --- | --- | --- | --- | --- | --- | --- | --- | --- | --- | --- | --- | --- | --- | --- | --- | --- |
|  | HE | HP |  | HE | HP |  | HE | HP |  | HE | HP |  | HE | HP |  | HE | HP |  | HE | HP |  | HE | HP |  | HE | HP |
| 1 | 68 | 69 |  | 176.9±1.2 | 176.7±1.2 |  | 24.9±16.9 | 22.9±10.0 |  | 16.2±2.0 | 15.5±2.9 |  | 0.000±0.000 | 0.154±0.156 |  | 18.5±10.7 | 13.4±9.0 |  | 39.6±10.4 | 31.6±13.5 | | 782.1±533.9 | 506.8±478.5 | | 0.466 | 0.891 |
| 2 | 91 | 93 |  | 177.6±1.8 | 177.7±1.6 |  | 18.9±9.7 | 18.6±9.1 |  | 15.6±1.7 | 15.9±1.9 |  | 0.001±0.007 | 0.192±0.242 |  | 12.6±6.7 | 8.7±6.6 |  | 26.4±10.6 | 24.9±11.5 | | 376.4±298.4 | 257.8±224.7 | | 0.628 | 0.760 |
| 3 | 84 | 81 |  | 176.9±1.6 | 176.9±1.4 |  | 14.3±7.4 | 15.3±7.8 |  | 15.8±2.0 | 15.5±2.0 |  | 0.003±0.023 | 0.218±0.213 |  | 10.1±6.8 | 8.1±6.4 |  | 25.1±11.1 | 26.8±12.6 | | 290.6±257.0 | 266.1±282.6 | | 0.782 | 1.128 |
| 4 | 97 | 93 |  | 176.5±1.7 | 176.2±2.2 |  | 18.1±9.6 | 17.5±9.2 |  | 15.9±2.2 | 15.5±1.9 |  | 0.000±0.000 | 0.269±0.205 |  | 12.6±8.7 | 9.2±7.6 |  | 29.4±12.9 | 28.5±13.0 | | 442.4±405.5 | 325.6±348.4 | | 0.840 | 1.145 |
| 5 | 53 | 57 |  | 199.5±4.1 | 198.5±4.7 |  | 18.0±8.3 | 16.7±7.7 |  | 17.1±2.5 | 17.1±2.0 |  | 0.000±0.000 | 0.320±0.232 |  | 10.6±5.4 | 7.2±7.1 |  | 28.9±8.9 | 26.9±16.2 | | 320.8±212.0 | 263.4±285.4 | | 0.437 | 1.174 |
| 6 | 80 | 87 |  | 199.6±3.9 | 198.5±5.6 |  | 19.6±9.7 | 16.7±7.4 |  | 16.2±2.5 | 16.8±2.3 |  | 0.002±0.012 | 0.321±0.196 |  | 11.7±7.7 | 6.3±5.5 |  | 31.1±10.5 | 25.3±14.1 | | 421.0±378.9 | 215.3±247.8 | | 0.810 | 1.324 |
| 7 | 90 | 88 |  | 176.3±1.7 | 176.2±1.4 |  | 16.3±8.1 | 17.0±8.1 |  | 16.3±2.6 | 16.2±2.1 |  | 0.000±0.000 | 0.422±0.220 |  | 12.5±6.9 | 7.9±6.2 |  | 32.6±9.4 | 27.3±10.8 | | 434.1±322.5 | 254.2±281.8 | | 0.552 | 1.229 |
| 8 | 79 | 87 |  | 176.8±1.5 | 176.9±1.4 |  | 20.2±10.2 | 18.5±9.9 |  | 15.7±2.4 | 16.3±2.3 |  | 0.011±0.031 | 0.445±0.284 |  | 11.6±8.5 | 8.4±7.9 |  | 32.3±10.2 | 28.0±14.5 | | 425.4±381.1 | 294.7±341.0 | | 0.802 | 1.339 |
| 9 | 101 | 116 |  | 201.1±2.9 | 201.2±2.6 |  | 17.8±7.5 | 17.7±6.3 |  | 14.8±2.2 | 14.4±2.3 |  | 0.001±0.005 | 0.462±0.169 |  | 11.6±7.4 | 6.4±4.9 |  | 33.8±12.4 | 28.2±15.3 | | 446.3±407.3 | 233.7±264.1 | | 0.833 | 1.277 |
| 10 | 63 | 60 |  | 180.6±2.1 | 180.8±1.6 |  | 18.2±10.4 | 23.8±14.6 |  | 14.9±2.6 | 14.8±2.4 |  | 0.003±0.016 | 0.550±0.239 |  | 10.5±8.1 | 7.0±6.7 |  | 30.1±10.7 | 26.1±11.8 | | 356.3±320.8 | 227.6±277.1 | | 0.811 | 1.482 |
| 11 | 87 | 89 |  | 176.8±1.7 | 177.0±1.4 |  | 21.6±10.8 | 21.3±6.4 |  | 15.3±2.3 | 15.5±1.5 |  | 0.001±0.006 | 0.557±0.221 |  | 13.6±8.9 | 8.4±6.3 |  | 38.3±11.2 | 25.6±12.3 | | 566.4±473.4 | 267.8±262.8 | | 0.699 | 0.963 |

**TABLE S5** Differences in net directional selection on floral traits among populations of *Primula florindae* using ANCOVA.

| Terms | *df* | *SS* | *MS* | *F* | *P* |
| --- | --- | --- | --- | --- | --- |
| Population | 10 | 0 | 0 | 0 | 1 |
| Flowering start date | 1 | 22 | 22 | 45.272 | **< 0.001** |
| Number of flowers | 1 | 579.1 | 579.1 | 1189.98 | **< 0.001** |
| Corolla size | 1 | 1.8 | 1.8 | 3.648 | 0.056 |
| Population × flowering start date | 10 | 10.4 | 1 | 2.137 | **0.02** |
| Population × number of flowers | 10 | 3.1 | 0.3 | 0.646 | 0.775 |
| Population × corolla size | 10 | 4.1 | 0.4 | 0.848 | 0.582 |
| Residuals | 876 |  |  |  |  |

**TABLE S6** Directional selection gradients (β_i_ ± SE) and associated *P*-values among herbivore-present treatment (HP) and herbivore-exclusion treatment (HE) in 11 *Primula florindae* populations. Herbivore-mediated selection (Δβ_herb_ = β_HP_ - β_HE_) and *P*-values associated with the trait × herbivory treatment interactions in ANCOVAs conducted separately for each population are also given. Significant selection estimates and their *P*-values are indicated in bold.

| Traits, by site | HP | | |  | | HE | | |  | | Herbivore-mediated selection | | |
| --- | --- | --- | --- | --- | --- | --- | --- | --- | --- | --- | --- | --- | --- |
|  | β_i_ ± SE | *P* |  | | β_i_ ± SE | | *P* |  | | Δβ_herb_ ± SE | | *P* |  |
| Population 1 |  |  |  | |  | |  |  | |  | |  |  |
| Flowering start date | -0.062±0.084 | 0.468 |  | | 0.032±0.028 | | 0.258 |  | | -0.094±0.089 | | 0.286 |  |
| Number of flowers | **0.712±0.091** | **< 0.001** |  | | **0.633±0.031** | | **< 0.001** |  | | 0.079±0.096 | | 0.132 |  |
| Corolla size | 0.094±0.091 | 0.302 |  | | 0.017±0.029 | | 0.574 |  | | 0.077±0.096 | | 0.403 |  |
| Population 2 |  |  |  | |  | |  |  | |  | |  |  |
| Flowering start date | 0.010±0.063 | 0.879 |  | | -0.015±0.046 | | 0.744 |  | | 0.025±0.078 | | 0.847 |  |
| Number of flowers | **0.664±0.060** | **< 0.001** |  | | **0.688±0.044** | | **< 0.001** |  | | -0.024±0.074 | | 0.782 |  |
| Corolla size | 0.023±0.064 | 0.713 |  | | -0.001±0.045 | | 0.974 |  | | 0.024±0.078 | | 0.75 |  |
| Population 3 |  |  |  | |  | |  |  | |  | |  |  |
| Flowering start date | 0.032±0.079 | 0.684 |  | | 0.012±0.054 | | 0.817 |  | | 0.02±0.096 | | 0.83 |  |
| Number of flowers | **0.845±0.075** | **< 0.001** |  | | **0.761±0.054** | | **< 0.001** |  | | 0.084±0.092 | | 0.355 |  |
| Corolla size | -0.044±0.081 | 0.585 |  | | -0.039±0.056 | | 0.488 |  | | -0.005±0.098 | | 0.96 |  |
| Population 4 |  |  |  | |  | |  |  | |  | |  |  |
| Flowering start date | **0.210±0.074** | **0.006** |  | | 0.080±0.070 | | 0.253 |  | | 0.13±0.102 | | 0.094 |  |
| Number of flowers | **0.734±0.076** | **< 0.001** |  | | **0.579±0.070** | | **< 0.001** |  | | 0.155±0.103 | | 0.168 |  |
| Corolla size | 0.078±0.069 | 0.267 |  | | **0.147±0.071** | | **0.04** |  | | -0.069±0.099 | | 0.487 |  |
| Population 5 |  |  |  | |  | |  |  | |  | |  |  |
| Flowering start date | **0.224±0.086** | **0.012** |  | | 0.039±0.058 | | 0.503 |  | | **0.185±0.104** | | **0.015** |  |
| Number of flowers | **0.864±0.085** | **< 0.001** |  | | **0.543±0.054** | | **< 0.001** |  | | **0.321±0.101** | | **0.003** |  |
| Corolla size | -0.042±0.086 | 0.626 |  | | 0.024±0.057 | | 0.667 |  | | -0.066±0.103 | | 0.532 |  |
| Population 6 |  |  |  | |  | |  |  | |  | |  |  |
| Flowering start date | **0.203±0.076** | **0.009** |  | | -0.011±0.062 | | 0.861 |  | | **0.214±0.098** | | **0.01** |  |
| Number of flowers | **0.856±0.083** | **< 0.001** |  | | **0.684±0.062** | | **< 0.001** |  | | 0.172±0.104 | | 0.14 |  |
| Corolla size | 0.086±0.083 | 0.306 |  | | **0.165±0.064** | | **0.012** |  | | -0.079±0.105 | | 0.457 |  |
| Population 7 |  |  |  | |  | |  |  | |  | |  |  |
| Flowering start date | 0.061±0.080 | 0.445 |  | | -0.015±0.036 | | 0.68 |  | | 0.076±0.088 | | 0.403 |  |
| Number of flowers | **0.792±0.102** | **< 0.001** |  | | **0.617±0.036** | | **< 0.001** |  | | 0.175±0.108 | | 0.089 |  |
| Corolla size | 0.078±0.100 | 0.44 |  | | **0.126±0.037** | | **< 0.001** |  | | -0.048±0.107 | | 0.633 |  |
| Population 8 |  |  |  | |  | |  |  | |  | |  |  |
| Flowering start date | -0.061±0.093 | 0.509 |  | | **-0.132±0.060** | | **0.031** |  | | 0.071±0.111 | | 0.438 |  |
| Number of flowers | **0.781±0.093** | **< 0.001** |  | | **0.592±0.064** | | **< 0.001** |  | | 0.189±0.113 | | 0.078 |  |
| Corolla size | **0.209±0.094** | **0.029** |  | | **0.213±0.068** | | **0.003** |  | | -0.004±0.116 | | 0.98 |  |
| Population 9 |  |  |  | |  | |  |  | |  | |  |  |
| Flowering start date | 0.076±0.077 | 0.328 |  | | 0.036±0.061 | | 0.551 |  | | 0.04±0.098 | | 0.395 |  |
| Number of flowers | **0.806±0.075** | **< 0.001** |  | | **0.749±0.051** | | **< 0.001** |  | | 0.057±0.091 | | 0.639 |  |
| Corolla size | 0.018±0.078 | 0.819 |  | | 0.098±0.061 | | 0.113 |  | | -0.08±0.099 | | 0.448 |  |
| Population 10 |  |  |  | |  | |  |  | |  | |  |  |
| Flowering start date | 0.071±0.132 | 0.593 |  | | 0.001±0.046 | | 0.987 |  | | 0.07±0.140 | | 0.351 |  |
| Number of flowers | **0.821±0.147** | **< 0.001** |  | | **0.792±0.049** | | **< 0.001** |  | | 0.029±0.155 | | 0.664 |  |
| Corolla size | -0.077±0.150 | 0.609 |  | | 0.092±0.052 | | 0.084 |  | | -0.169±0.159 | | 0.28 |  |
| Population 11 |  |  |  | |  | |  |  | |  | |  |  |
| Flowering start date | -0.049±0.063 | 0.436 |  | | -0.051±0.050 | | 0.307 |  | | 0.002±0.080 | | 0.92 |  |
| Number of flowers | **0.804±0.066** | **< 0.001** |  | | **0.700±0.051** | | **< 0.001** |  | | 0.104±0.083 | | 0.221 |  |
| Corolla size | -0.011±0.067 | 0.876 |  | | 0.013±0.051 | | 0.795 |  | | -0.024±0.084 | | 0.777 |  |

**TABLE** **S7** Goodness-of-fit statistics for models of the relations between opportunity for selection, strength of herbivore-mediated selection and herbivory intensity among 11 *Primula florindae* populations.

| Terms | Models | AIC/DIC | *P* value of the hypothesis model | *P* value of each predictor (linear and quadratic terms) |  | Terms | Models | AIC/DIC | *P* value of the hypothesis model | *P* value of each predictor (linear and quadratic terms) | |
| --- | --- | --- | --- | --- | --- | --- | --- | --- | --- | --- | --- |
| Opportunity for selection | | | | | | | | | | |  |
| HP treatment | A | -2.449 | 0.059 | 0.059 |  | HE treatment | A | -7.537 | 0.191 | 0.191 | |
|  | B | **-4.888** | **0.038** | **0.081** |  |  | B | -9.379 | 0.111 | 0.105 | |
| Strength of herbivore-mediated directional selection | | | | | | | | | | |  |
| Flowering start date | C | -33.323 |  | 0.794 |  | Number of flowers | C | -25.766 |  | 0.726 | |
|  | D | -34.026 |  | 0.114 |  |  | D | **-28.069** |  | **0.042** | |
| Corolla size | C | -43.99 |  | 0.376 |  |  |  |  |  |  | |
|  | D | -41.788 |  | 0.938 |  |  |  |  |  |  | |

Notes: Model A is the OLS linear regression model, including the linear term of herbivory intensity as the explanatory variable; model B is the OLS nonlinear regression model, including both the linear and quadratic terms of herbivory intensity as the explanatory variables; model C is the Bayesian model, including the linear term of herbivory intensity as the explanatory variable and model D is the Bayesian model, including the linear and quadratic terms of herbivory intensity as the explanatory variables. Each model represents an alternative hypothesis for the relations between opportunity for selection, strength of herbivore-mediated selection and the intensity of herbivory. The model with lowest AIC or DIC value provides a better fit to the data. The AIC value is for models A and B, and the DIC value is for models C and D. The HP treatment represents the herbivore-present treatment, the HE treatment represents the herbivore-exclusion treatment.
